# Adapting the KAER Framework to Support Early Diagnosis and Treatment of Dementia in Asian Americans

**DOI:** 10.1093/geroni/igaa057.2851

**Published:** 2020-12-16

**Authors:** Tina Sadarangani, Jennifer Zanowiak, Janet Pan, Vanessa Salcedo, Stella Yi, Simona Kwon, Joshua Chodosh, Chau Trinh-Shevrin

**Affiliations:** 1 New York University, New York, New York, United States; 2 NYU Grossman School of Medicine, New York, New York, United States; 3 NYU School of Medicine, New York, New York, United States; 4 New York University Grossman School of Medicine, New York, New York, United States

## Abstract

Asian Americans (AAs) are frequently diagnosed with dementia in advanced disease stages and have difficulty accessing services. The NYU Center for the Study of Asian American Health, set out to culturally adapt The Kickstart-Assess-Evaluate-Refer (KAER) framework to support earlier detection of dementia in AA communities. Working with Bangladeshi, Chinese, and Korean senior centers, we used a participatory action approach to assess cultural relevancy, usability and acceptability of KAER to improve timely diagnosis and access to care. We found that community-based organizations (CBOs), not physicians, were often “first responders” in identifying and managing dementia. However, CBO staff felt unprepared to “Kickstart” discussions, found certain KAER questions were not culturally appropriate, and encountered barriers in communicating their concerns to physicians. Adaptations to KAER can maximize its impact and reach in AA communities. Suggestions include group education, as opposed to individualized screening, and stronger linkages between physicians and CBOs to ensure care continuity.

